# The Role of Hemicellulose in Cadmium Tolerance in Ramie (*Boehmeria nivea* (L.) Gaud.)

**DOI:** 10.3390/plants11151941

**Published:** 2022-07-26

**Authors:** Yushen Ma, Hongdong Jie, Yanyi Tang, Hucheng Xing, Yucheng Jie

**Affiliations:** 1 College of Agronomy, Hunan Agricultural University, Changsha 410128, China; mys9204@stu.hunau.edu.cn (Y.M.); jhd20210218@stu.hunau.edu.cn (H.J.); 1845552150@stu.hunau.edu.cn (Y.T.); xinghucheng@hunau.edu.cn (H.X.); 2 Hunan Provincial Key Laboratory of Crop Germplasm Innovation and Utilization, Changsha 410128, China

**Keywords:** hemicellulose, cadmium, ramie, tolerance

## Abstract

Ramie cell walls play an important role in cadmium (Cd) detoxification. However, the Cd binding capacity of the cell wall components and the cell wall compositions among ramie species remains unclear. Therefore, this study compared two ramie populations (‘Dazhuhuangbaima’ (low-Cd-accumulating population) and ‘Zhongzhu 1’ (high-Cd-accumulating population)) with different Cd enrichment characteristics. The two ramie populations were treated with 0, 25, and 75 mg kg^−1^ Cd for 30 days; then, their root length, plant height, biomass, Cd enrichment in the organs, subcellular Cd distribution, Cd content in the cell wall polysaccharides, and hemicellulose content were determined. The root length, plant height, biomass, and Cd enrichment in all organs were significantly higher (*p* ≤ 0.05) in ‘Zhongzhu 1’ than in ‘Dazhuhuangbaima’ under Cd stress. In addition, the subcellular Cd distribution analysis revealed that Cd was mainly found in the cell wall in both ramie populations. Among the cell wall fractions, Cd was mainly bound to the hemicelluloses, with 60.38–73.10% and 50.05–64.45% Cd accumulating in the ‘Zhongzhu 1’ and ‘Dazhuhuangbaima’ cell wall hemicelluloses, respectively. However, the Cd concentration in the ‘Zhongzhu 1’ hemicellulose was significantly higher (*p* ≤ 0.05) than that in the ‘Dazhuhuangbaima’ hemicellulose. Hemicellulose content analysis further revealed that the hemicellulose concentration increased with the Cd concentration in both populations, but it was significantly higher (*p* ≤ 0.05) in ‘Zhongzhu 1’ than in ‘Dazhuhuangbaima’ across all Cd treatments. Thus, ramie copes under Cd stress by increasing the hemicellulose content in the cell wall. The findings in this study confirm that hemicellulose is the main enrichment site for Cd in ramie. It also provides a theoretical basis for Cd enrichment breeding in ramie.

## 1. Introduction

Over the past 50 years, heavy metals have seriously polluted the environment [1], resulting in people growing crops on farmlands with a widespread accumulation of heavy metals in the soil [2]. For example, high concentrations of heavy metals, including plumbum (Pb) and cadmium (Cd), have been detected in vegetables and fruits grown in Ginfel River near Sheba Tannery, Tigray, Northern Ethiopia [3], and in vegetables grown in Vadodara, Gujarat, India [4]. In Bangladesh, the chromium (Cr), arsenic (As), Cd, and Pb levels in food crops around the industrial areas are higher than the maximum standards [5]. There have been “Cd rice” accidents in southern China [6]. Heavy metal contamination in crops enters the human body through the consumption of contaminated foods such as rice [7] and vegetables [8], posing a severe threat to human health. Thus, the presence of heavy metals in the soil has become a significant environmental problem globally.

Cd is the seventh most toxic heavy metallic element widely found in the earth’s crust, per the Agency for Toxic Substances and Disease Registry (ATSDR) ranking [9]. In China, more than 13,000 hectares, consisting of 25 regions in 11 provinces, and more than 1.46 × 10^8^ kg/annum of agricultural products from these areas are polluted with Cd [10,11]. Therefore, the severity of Cd-contamination poses a major public health threat, which requires immediate action.

Excess Cd affects the physiological metabolisms of plants, including root growth [12], photosynthesis rate [13], transpiration [14], and leaf chlorosis [15]. The Cd subcellular distribution affecting the Cd migration and toxicity is an important mechanism by which plants cope with Cd stress. In addition, the cell wall plays a significant role in Cd tolerance and accumulation by preventing Cd migration into the cell and binding the cell area. For example, in *Kandelia obovata*, more than 54% of Cd was localized in the cell wall, with a low amount penetrating the cell organelles [16]. Fractionation and ultrastructural localization studies have also revealed that most Cd is accumulated in the cell wall in *Dittrichia viscosa* [17]. Scanning Electron Microscopy (SEM) and Transmission Electron Microscopy (TEM) have also revealed that Cd is mainly distributed in the cell wall in *Ceratopteris pteridoides* [18] and *Microsorum pteropus* [19]. Further, 48.2 to 61.9% of Cd accumulated by ramie is localized in the cell wall [20]. Cd is also mainly distributed in the cell walls of *Oryza sativa* [21] and *Triticum aestivum* [22]; hence, it is a key phenomenon in both dicotyledons and monocotyledons.

The cell wall comprises celluloses, hemicelluloses, pectins, lignin, and enzymatic and structural proteins [23,24,25]. The ability of the cell wall to bind Cd depends on functional groups, such as -NH_2_, -COOH, -OH, and -SH present in cell wall polysaccharides [26,27]. Hemicellulose responds fastest to cuprum (Cu) stress, binding more than 80% of the total Cu in the cell wall of *Ricinus communis* under Cu stress [28]. The cell wall hemicellulose has been recognized as the major target of aluminum (Al) and Cd accumulation in *Arabidopsis* [29,30] and Al in *Oryza sativa* [31]. Pectins have also been reported as a major polysaccharide binding Cd in *Brassica chinensis*, with up to 79.4% of cell wall-bound Cd in pectins [32]. More than half of Cd in the root cell wall of a low-Cd-accumulating soybean was accumulated in the pectin, but more than half of Cd in the root cell wall of high-Cd soybean was accumulated in the cellulose [33]. However, the cell wall components key to the Cd enrichment of ramie remain unclear.

Phytoremediation is a new technology for removing heavy metals from the soil using plants. Unfortunately, many Cd hyperaccumulators exhibit a slow growth rate, low biomass, and limited effectiveness in removing Cd from the soil [34]. Ramie (*Boehmeria nivea*) is a perennial herb with rapid growth accumulating high biomass. Previous studies revealed that ramie is a potential crop for the phytoremediation of Cd-contaminated soils [35] by accumulating Cd in the cell wall [20]. However, the mechanism of Cd retention in the cell wall components of ramie remains unclear. Therefore, this study aimed to analyze and determine the cell wall components key to Cd tolerance and enrichment. Two ramie populations with different Cd enrichment characteristics were selected, and the root length, plant height, biomass, Cd enrichment in the organs, subcellular Cd distribution, amount of Cd in the cell wall polysaccharide, and hemicellulose concentration in the two populations were determined. To investigate the role of cell wall polysaccharides in the Cd enrichment of ramie and to identify the major polysaccharide for Cd binding, we explored the intrinsic mechanism of Cd uptake in the cell walls fraction and the adaptive physiological mechanism of Cd accumulation in ramie cell walls. The findings in this study provide a theoretical basis for the Cd enrichment breeding of ramie.

## 2. Results

### 2.1. Effects of Cd on Plant Growth

**Figure 1:**
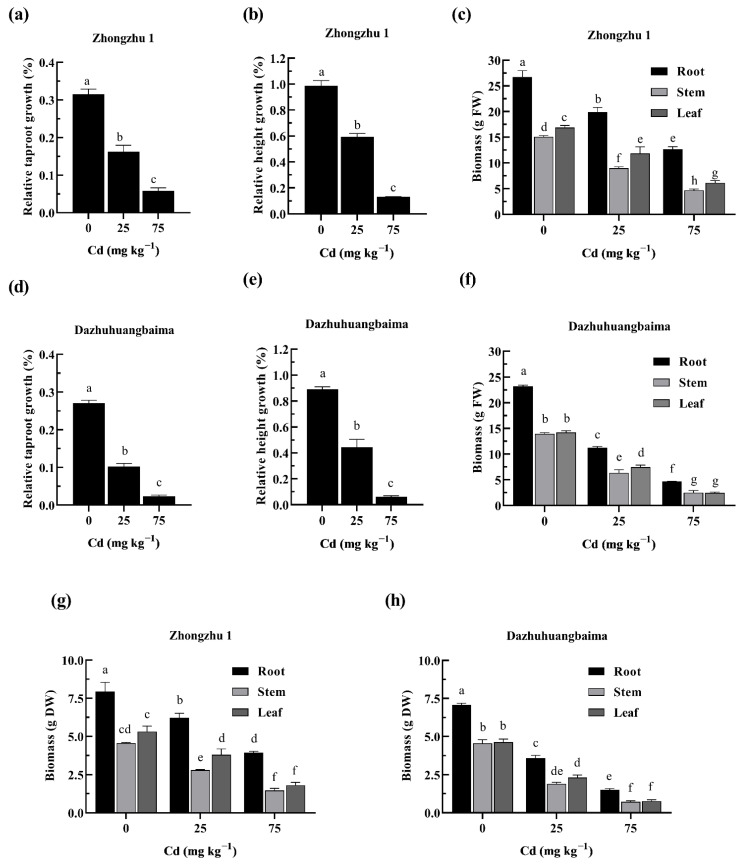
The effect of Cd on the taproot length, plant height, and biomass of ramie. (**a**) The relative taproot growth of ‘Zhongzhu 1’. (**b**) The relative height growth of ‘Zhongzhu 1’. (**c**) The fresh weight (FW) of ‘Zhongzhu 1’. (**d**) The relative taproot growth of ‘Dazhuhuangbaima’. (**e**) The relative height growth of ‘Dazhuhuangbaima’. (**f**) The FW of ‘Dazhuhuangbaima’. (**g**) The dry weight (DW) of ‘Zhongzhu 1’. (**h**) The DW of ‘Dazhuhuangbaima’. Data presented as means ± SD (*n* = 18). Different letters indicate significant differences at *p* ≤ 0.05.

Cd treatment on ramie cuttings for 30 days inhibited the increase in taproot length and plant height. Compared to the control, the 25 mg kg^−1^ Cd inhibited the increase in the taproot length of ‘Zhongzhu 1’ and ‘Dazhuhuangbaima’ by 48.46% and 61.99%, while the plant height was inhibited by 39.84% and 50.19%, respectively. With the 75 mg kg^−1^ Cd, the increase in taproot length was inhibited by 81.46% and 91.27%, while the plant height was inhibited by 86.82% and 93.26% in ‘Zhongzhu 1’ and ‘Dazhuhuangbaima’, respectively (Figure 1a,b,d,e). In addition, the roots, stems, and leaves biomass of ‘Zhongzhu 1’ plants treated with 25 mg kg^−1^ Cd were decreased by 25.46, 40.51, and 29.76%, respectively, and 52.56, 69.00, and 63.47%, respectively, following treatment with 75 mg kg^−1^ Cd (Figure 1c,g). At the same time, treatment with 25 mg kg^−1^ Cd decreased the roots, stems, and leaves biomass of ‘Dazhuhuangbaima’ by 51.38, 54.51, and 47.52%, while 75 mg kg^−1^ Cd decreased the roots, stems, and leaves biomass by 79.86, 82.04, and 82.68%, respectively (Figure 1f,h). Overall, the inhibitory effect against root growth and plant height was more pronounced with increasing Cd concentrations. However, the taproot length, plant height, fresh weights (FW) and dry weight (DW) were significantly higher in ‘Zhongzhu 1’ than in ‘Dazhuhuangbaima’ (*p* ≤ 0.05) across all treatments.

### 2.2. Cd Accumulation in Ramie under Cd Treatment

**Figure 2:**
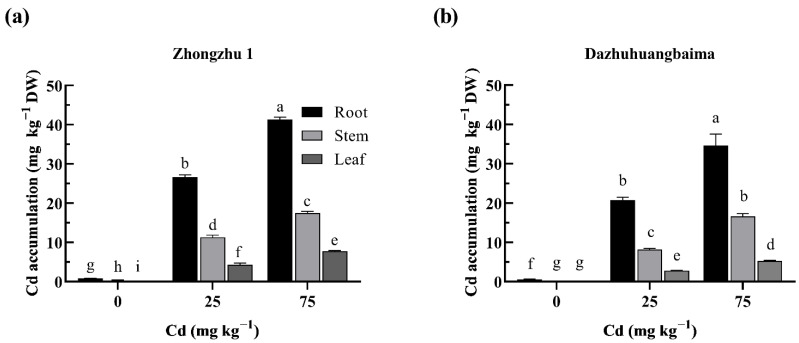
Cd accumulation in the roots, stems, and leaves of ‘Zhongzhu 1’ (**a**) and ‘Dazhuhuangbaima’ (**b**) under different Cd concentrations. DW, dry weight. Data represent means ± SD (*n* = 3). Different letters indicate significant differences at *p* ≤ 0.05.

The Cd contents in different ramie tissues following 30-day Cd exposure are shown in Figure 2. The Cd concentrations in the roots, stems, and leaves were increased with the increase in Cd content in the soil. However, the Cd content was approximately 61.31–65.31% in the roots, 25.80–29.39% in the stems, and 8.89–11.60% in the leaves. In addition, the Cd content in the ‘Zhongzhu 1’ organs was significantly higher (*p* ≤ 0.05) than that in ‘Dazhuhuangbaima’ across all treatments. However, the Cd translocation factors were higher in ‘Dazhuhuangbaima’ than in ‘Zhongzhu 1’.

### 2.3. Subcellular Cd Distribution

A comparison among the Cd contents in the cell walls, organelles, and soluble fractions in the two ramie populations revealed that the Cd content increased with an increase in the Cd concentration in the soil (Table 1). Most of the Cd was accumulated in the cell walls fraction, with 45.65–71.93% of the total Cd. Across all treatments, the proportion of Cd enrichment in the cell walls of ‘Zhongzhu 1’ was higher than that of ‘Dazhuhuangbaima’.

### 2.4. Hemicellulose Was the Major Target of Cd

**Figure 3:**
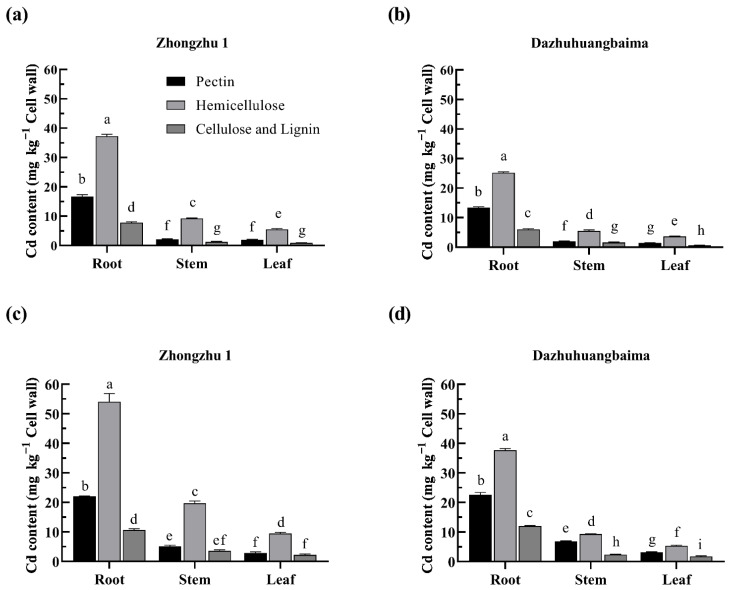
Cd content in the cell wall polysaccharides of two ramie populations. Cd content in the cell wall polysaccharides of ‘Zhongzhu 1’ treated with (**a**) 25 mg kg^−1^ and (**c**) 75 mg kg^−1^ Cd, respectively, and ‘Dazhuhuangbaima’ treated with (**b**) 25 mg kg^−1^ and (**d**) 75 mg kg^−1^ Cd, respectively. Data represent means ± SD (*n* = 3). Different letters indicate significant differences at *p* ≤ 0.05.

The Cd contents in the different cell wall polysaccharides are shown in Figure 3. The Cd concentration in the different cell wall fractions increased with the increase in the Cd concentration in the soil. Across all treatments, hemicellulose accumulated the most Cd, with 56.46% of the total cell-wall-accumulated Cd. The Cd bound to the ‘Zhongzhu 1’ hemicellulose was 1.4–2.1 times that of ‘Dazhuhuangbaima’. In addition, there were slight differences in the Cd accumulated in the celluloses in the two ramie populations.

### 2.5. The Link between Hemicellulose Content and Cd Accumulation

**Figure 4:**
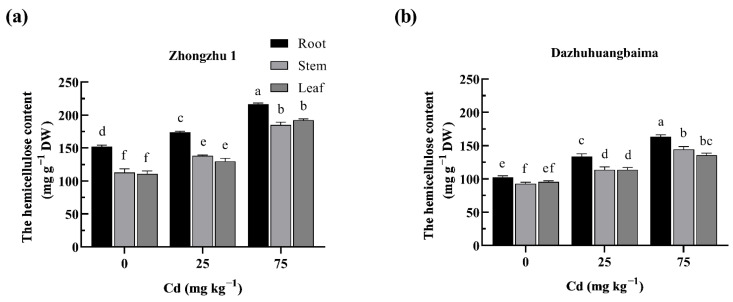
The hemicellulose contents in two ramie populations under Cd stress. The hemicellulose contents in: (**a**) ‘Zhongzhu 1’ treated with 0, 25, and 75 mg kg^−1^ Cd; (**b**) ‘Dazhuhuangbaima’ treated with 0, 25, and 75 mg kg^−1^ Cd. DW, Dry weight. Data represent means ± SD (*n* = 3). Different letters indicate significant differences at *p* ≤ 0.05.

The hemicellulose contents in the cell walls of the two ramie populations are presented in Figure 4. The hemicellulose contents were increased with the increase in the Cd concentration in the soil. Across all treatments, the hemicellulose contents in ‘Zhongzhu 1’ were significantly higher (*p* ≤ 0.05) than those in ‘Dazhuhuangbaima’.

**Figure 5:**
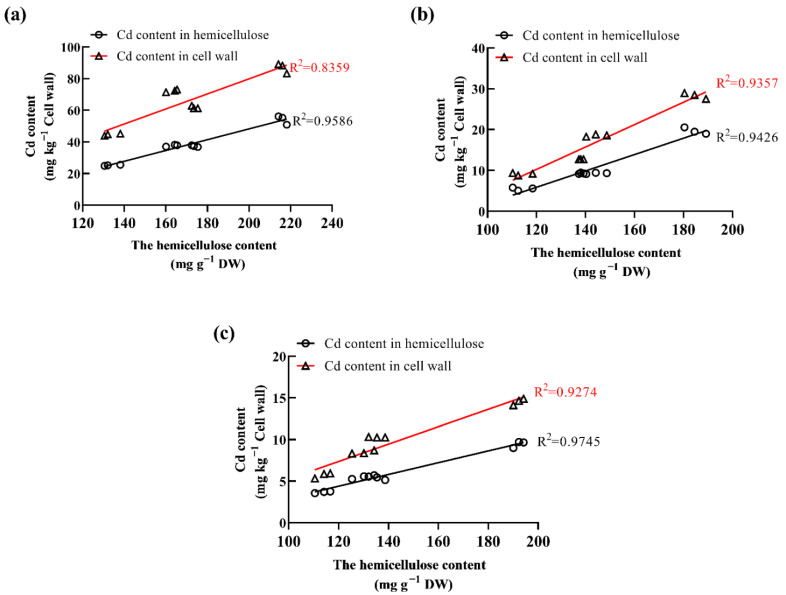
Correlations between the hemicellulose content and Cd concentration in the: (**a**) root; (**b**) stem; (**c**) leaf.

Correlations analysis revealed that the hemicellulose content was positively (*p* ≤ 0.05) related with the Cd concentration in the hemicellulose and cell wall (Figure 5). Thus, Cd promoted an increase in the hemicellulose content in the ramie cell walls, thereby resisting Cd toxicity.

## 3. Discussion

To survive in Cd-contaminated soil, plants use a wide range of defense mechanisms, including Cd binding to the cell wall, Cd exclusion, the synthesis of phytochelatins, Cd compartmentalization in vacuoles, and the synthesis of metallothioneins or stress proteins [36]. In this study, the ramie cell wall was the first barrier protecting the cells from Cd toxicity, with more than half of the Cd concentrating in the cell wall. A similar phenomenon has been reported in *Lactuca* [37]. In contrast, in *Brassica napus* and *Phytolacca americana*, less Cd is retained at the cell wall, with most of the Cd being accumulated as a soluble fraction [38,39]. These differences may be attributed to the different tolerance strategies employed by different plants under Cd stress. The vacuole is also a major organ-accumulating Cd [40] and an important storage site for excess metals following their chelation by cytosolic ligands [41]. In this study, the content of Cd in the soluble fraction increased with an increase in the Cd content in the soils. This is because the Cd retention in the cell walls was limited, whereas the Cd concentration increased in the soil; thus, Cd accumulated in the soluble fraction to reduce Cd in the cells, reducing the Cd toxicity. Similar results were reported in *Brassica chinensis* [32]. Moreover, in this study, the Cd concentration in the cell wall of ‘Zhongzhu 1’ (high-Cd-accumulating population) was higher than that in ‘Dazhuhuangbaima’ (low-Cd accumulating population). However, in *Brassica chinensis*, more Cd was bound to the cell wall fraction of low-Cd-accumulating cultivar than to the high-Cd cultivar [32].

Different cell wall components have different Cd binding capacities, with pectins and hemicelluloses playing key roles in Cd binding [41]. Guo et al. [42] revealed that -COOH and -OH in hemicelluloses bind heavy metals, with hemicelluloses containing xyloglucans, xylans, and glucomannans binding Cd. In the study, approximately 60% of the total Cd in the cell wall was accumulated in the hemicelluloses, followed by pectins. These results validate that hemicelluloses and pectins are the main Cd accumulators in the ramie cell wall, with hemicellulose being superior to pectins in binding Cd. This is consistent with previous reports on *Elsholtzia splendens* [43] and some rice varieties [44], but it contradicts reports in willow [45].

The responses of cell wall polysaccharides to heavy metal stress among different cultivars are different. In soybean, Cd is mainly concentrated in pectin in the root cell wall of the low-Cd-accumulating variety but in the cellulose in the high-Cd accumulating variety [33]. In this study, ‘Zhongzhu 1’ had a significantly higher Cd concentration in the hemicellulose fractions than ‘Dazhuhuangbaima’. Similarly, in rice, the Cd bound to the hemicelluloses in the root cell wall of Cd-tolerant cultivars is higher than that in sensitive cultivars [44]. The ability of cell walls to bind Cd is related to its structural changes [46]. Thus, the differences between the two ramie populations may be related to the changes in the structure and content of cell wall components following exposure to Cd stress.

The cell wall comprises 80–90% polysaccharides [47]. The content of the cell wall polysaccharides is calculated based on the glucose or uronic acid concentrations [48]. Plant cell walls contain many -COOH and -OH, which bind metal cations [49]. The plant cell walls are actively remodeled to increase the content of cell wall components, thereby enhancing the accumulation of heavy metals [50,51]. Therefore, the changes in hemicellulose composition affect the plant resistance to trace metals [52]. In this study, the hemicellulose contents in both ramie populations were significantly increased under Cd stress, consistent with previous reports in *Sedum alfredii* [53] and wheat [54] under Cd stress. Similar phenomena have been observed in other heavy metal stresses. For example, pectin and hemicellulose contents in the wheat root cell wall are significantly increased under Al stress [55]. Under Cu stress, the total sugars and uronic acids in pectin, hemicellulose, and cellulose in *Chrysanthemum coronarium* are significantly increased [56]. The concentrations of hemicellulose and Cd enriched in the hemicellulose and cell wall in ‘Zhongzhu 1’ were also higher than those in ‘Dazhuhuangbaima’. This is consistent with previous studies reporting that, in rice roots with low pectin and hemicellulose levels, the Cd fixation in the cell wall was low [48]. Thus, hemicelluloses were the main enrichment site for Cd in ramie. The higher hemicellulose concentrations in ‘Zhongzhu 1’ indicate that there are heavier metal-binding sites, which absorb more Cd; hence, there is a higher Cd tolerance and enrichment in ‘Zhongzhu 1’ than in ‘Dazhuhuangbaima’. The modifications of the hemicellulose polysaccharides, such as acetylation [57] and fucosylation [58], enhance the accumulation of heavy metals in plants. However, the existence of hemicellulose polysaccharide modification in ‘Zhongzhu 1’ and ‘Dazhuhuangbaima’ should be evaluated in further studies.

## 4. Materials and Methods

### 4.1. Plant Growth and Cd Treatment

Two ramie species—‘Dazhuhuangbaima’ (low-Cd-accumulating population) and ‘Zhongzhu 1’ (high-Cd-accumulating population)—were provided by the Hunan Agricultural University, Changsha, China. Ramie cuttings of uniform size (10 cm long) were planted in stainless steel pots (45 × 50 × 50 cm^3^; six plants per pot) containing soil supplemented with 0, 25, and 75mg kg^−1^ cadmium chloride (CdCl_2_). The ramie plants were maintained under a 14/10 h day/night cycle and a light intensity of 20,000 lux. The soils used had an organic matter content, total nitrogen content, total phosphorus content, and total potassium content of 24.01, 1.65, 0.85, and 15.27 g kg^−1^, respectively, and a pH of 5.35.

Thirty days after Cd exposure, the ramie cuttings were uprooted and separated into roots, stems, and leaves. The fresh weights (FW), root length, and plant height were measured and recorded. Next, the leaves, stems, and roots were immersed in 20 mM Na_2_EDTA solution for 15 min to remove adhering Cd on their surface, followed by rinsing in three changes of deionized water. The leaves, stems, and roots were then assigned into two groups. One group was digested with HNO_3_:HClO_4_ (3:1, *v*/*v*), and the total amounts of Cd were determined using a flame atomic absorption spectrometer, while the other group was frozen in liquid N_2_ for the determination of the subcellular fractions and cell wall components.

### 4.2. Separation of Subcellular Fractions

The subcellular fractions were separated as described by Lai [59], with minor adjustments. The plant samples were homogenized in a mixture containing 250 mM sucrose, 50 mM Tris-HCI, and 1.0 mM DTT at 3000 rpm for 15 min at 4 °C, using an oscillator to obtain the cell wall fraction as the precipitate. The supernatant was centrifuged at 12,000 rpm for 30 min at 4 °C to obtain the cell organelle fraction as the precipitate and a soluble fraction containing some membranes, such as Golgi complex, endoplasmic reticulum, vesicles, tonoplast, and plasmalemma. Next, the cell wall fraction, organelle fraction, and soluble fraction were independently digested using HNO_3_:HClO_4_ (3:1, *v*/*v*), and the total amounts of Cd were determined using a flame atomic absorption spectrometer [60].

### 4.3. Cell Wall Extraction and Fractionation

The cell wall was extracted from the frozen plant materials following the protocol described by Wang et al. [33], with minor adjustments. The frozen plant materials were crushed in liquid N_2_ and homogenized in 75% cold ethanol, followed by an ice water bath for 20 min and centrifugation at 8000 rpm for 10 min at 4 °C. The precipitate was washed in two changes of acetone, methanol/chloroform (1:1, *v*/*v*), and methanol, respectively. The final precipitate containing the cell wall was freeze-dried and stored at 4 °C.

### 4.4. Extraction of Hemicelluloses, Celluloses, and Pectins from Ramie

Hemicelluloses, celluloses, and pectins were extracted from ramie cell walls as described by Li [53]. To extract pectins, the cell wall was incubated in deionized water in a boiling water bath for 1 h and then centrifuged at 16,800 rpm for 10 min at 4 °C. The supernatant solution contained pectin. The precipitate was further incubated in 4% NaOH to extract hemicellulose 1 (HC1) into the supernatant. Next, the resulting precipitate was incubated with 24% NaOH to extract hemicellulose 2 (HC2) into the supernatant, with only celluloses remaining in the precipitate. HC1 and HC2 collectively formed hemicelluloses. The samples were freeze-dried and stored at 4 °C, awaiting further use.

### 4.5. The Contents of Hemicellulose

The concentration of hemicelluloses was determined using the phenol sulfuric acid method [42]. Briefly, the hemicellulose extracts were incubated with 98% H_2_SO_4_ and 80% phenol at 100 °C for 15 min. After cooling to room temperature, their absorbance was determined by spectrophotometry at 490 nm.

### 4.6. Statistical Analysis

All data are presented as means ± SD. The differences between treatments were analyzed by one-way analysis of variance (ANOVA), followed by Tukey’s HSD post hoc test in SAS 9.4 software (SAS Institute, Cary, NC, USA) at a *p* ≤ 0.05 level of significance.

## 5. Conclusions

Ramie is a candidate for the phytoremediation of Cd-contaminated soils. Most of the Cd accumulated in the ramie cell wall is linked to hemicellulose. However, the hemicellulose content varies between the two ramie populations with different Cd tolerance capacities. A high hemicellulose content drives the high Cd tolerance and enrichment in the different ramie populations. Thus, hemicellulose plays an essential role in Cd tolerance. Overall, the findings in this study provide a theoretical basis for further research on the improvement of ramie to enhance its adaptability to Cd stress and its use in phytoremediation, including improving ramie hemicellulose levels, enhancing hemicellulose’s Cd enrichment ability, and enriching more Cd from the soil.

## Figures and Tables

**Table 1:** Subcellular Cd (mg kg^−1^ DW) distribution in different organs of two ramie populations.

Populations	Treatments	Organs	Cell Wall	Organelle	Soluble Fraction
‘Zhongzhu 1’		Root	16.08 ± 0.15a (60.28)	4.86 ± 0.30c (18.21)	5.73 ± 0.74b (21.50)
	25 mg kg^−1^	Stem	8.11 ± 0.10a (71.93)	1.39 ± 0.07c (12.33)	1.78 ± 0.69b (15.77)
		Leaf	3.03 ± 0.07a (70.72)	0.44 ± 0.12c (10.35)	0.81 ± 0.36b (18.88)
		Root	26.26 ± 0.14a (63.56)	5.57 ± 0.43c (13.47)	9.73 ± 0.50b (23.55)
	75 mg kg^−1^	Stem	11.02 ± 0.14a (62.99)	1.89 ± 0.10c (10.78)	4.59 ± 0.45b (26.22)
		Leaf	5.04 ± 0.06a (65.32)	0.95 ± 0.07c (12.28)	1.73 ± 0.35b (22.44)
‘Dazhuhuangbaima’		Root	9.95 ± 1.29a (48.11)	5.05 ± 0.08c (24.42)	5.68 ± 0.22b (27.47)
	25 mg kg^−1^	Stem	4.59 ± 0.16a (56.18)	1.82 ± 0.09b (22.28)	1.76 ± 0.13b (21.54)
		Leaf	1.66 ± 0.18a (59.19)	0.42 ± 0.08c (14.95)	0.73 ± 0.15b (25.88)
		Root	18.87 ± 0.37a (54.51)	6.31 ± 0.76c (18.23)	9.44 ± 0.28b (27.27)
	75 mg kg^−1^	Stem	8.88 ± 0.13a (53.46)	2.06 ± 0.05c (12.40)	5.67 ± 0.39b (34.14)
		Leaf	3.31 ± 0.20a (45.66)	1.86 ± 0.06b (25.66)	2.08 ± 0.33b (28.69)

DW, Dry weight. Data represent means ± SD (*n* = 3). Different letters indicate significant differences at *p* ≤ 0.05. The values in parentheses represent the proportion of Cd in the subcellular fraction of different organs.

## Data Availability

All data included in the main text.
